# Living Through the COVID‐19 Pandemic: The Experiences of People With Profound and Multiple Intellectual Disabilities Through a Family Carer Lens

**DOI:** 10.1111/jar.70088

**Published:** 2025-06-26

**Authors:** Dawn E. Cavanagh, Sue Caton, Jodie Rawles, Chris Hatton, Richard P. Hastings, the Coronavirus and People with Learning Disabilities Study Team, Richard Hastings, Chris Hatton, Jill Bradshaw, Sue Caton, Dawn Cavanagh, Amanda Gillooly, Andrew Jahoda, Rosemary Kelly, Roseann Maguire, Edward Oloidi, Jodie Rawles, Laurence Taggart, Stuart Todd

**Affiliations:** ^1^ Department of Social Care and Social Work Manchester Metropolitan University Manchester UK; ^2^ Intellectual Disabilities Research Institute (IDRIS) University of Birmingham Birmingham UK

**Keywords:** coronavirus, COVID‐19, family members, physical and mental health, profound and multiple intellectual disabilities, trauma

## Abstract

**Background:**

People with profound and multiple intellectual disabilities were disproportionately negatively affected by the COVID‐19 pandemic. Few studies exist about the perceived longer‐term impact of the pandemic on this group.

**Methods:**

Data were collected through a two‐stage narrative interview process. Six family members told their story about what their relative's lives were like at the first COVID‐19 ‘lockdown’, what their lives were like now, and what they hoped their lives would be like in the future. A second interview filled any gaps in the narrative. Data were analysed thematically.

**Results:**

Four themes were identified: Deeping exclusion and inequality; ‘I still haven't recovered’‐ Exhausted by the pandemic; A traumatic time–Disrupted routines, activities, and relationships and ‘The future is a scary place.’

**Conclusions:**

The COVID‐19 pandemic exacerbated pre‐existing health and social inequalities, leading to new uncertainties post‐pandemic. Addressing the existing deficit in service provision should be an urgent priority.

**Summary**

- It has been over 3 years since the COVID‐19 pandemic started.
- We wanted to hear more about what life had been like for people with profound and multiple intellectual disabilities and what life is like is now.
- We interviewed 6 family members of people with profound and multiple intellectual disabilities. We interviewed everyone twice, online.
- Families told us unfair lives got worse. People's daily lives and relationships changed. They told us it was hard to get healthcare and support during the pandemic and it is still hard now.

## 1 Introduction

Since the start of the COVID‐19 pandemic in March 2020, over 776.8 million confirmed COVID‐19 cases and over 7 million confirmed deaths were notified to WHO across 234 countries (World Health Organisation 2025). The majority of COVID‐19 associated deaths occurred between 2000 and 2022, with increased immunity leading to a significant decrease in deaths. However, long COVID continues to negatively impact human health and wellbeing, health systems, and the economy (Al‐Aly et al. 2024).

The first English lockdown (where people were ordered to ‘stay at home’) in response to the COVID‐19 pandemic began on 23rd March 2020 and lasted for over 3 months. This was followed by two further national lockdowns in 2021. Other restrictions included the ‘rule of six’—where indoor and outdoor social gatherings above six were banned; compulsory face mask wearing in most public venues and 2‐m social distancing. Most restrictions ended in June 2021 (Institute for Government Analysis 2024). People with profound and multiple intellectual disabilities were hit particularly hard by the pandemic, with the scaling down or removal of services and support greatly impacting this group due to the severity of their impairment and healthcare issues (Bradshaw et al. 2024).

The term ‘profound and multiple intellectual disabilities’ is typically used to describe a group of people with profound intellectual and multiple disabilities, limited verbal communication and often extreme physical and sensory disabilities and complex health needs (Profound and Multiple Learning Disabilities Network 2002). Health needs may include epilepsy, spasticity, pulmonary/respiratory problems, hearing problems, dysphagia, reflux disease and visual problems (Van Timmeren et al. 2016). People with profound and multiple intellectual disabilities will most likely require 24‐h‐a‐day support with all aspects of their lives including personal care, washing and dressing, eating, medical care, as well as ensuring that they have meaningful activity throughout the day.

While there has been a substantial body of research published in recent years that explores the experiences of people with intellectual disabilities during the COVID‐19 pandemic (e.g., Björnsdóttir 2024; Chadwick et al. 2023; Doody and Keenan 2021; Embregts et al. 2020; Lake et al. 2021), there is a gap in knowledge regarding the experiences of people with profound and multiple intellectual disabilities. Furthermore, there is a lack of literature which provides a COVID‐19 ‘retrospective’ and/or which considers the long‐term impacts of Covid‐19 for people with intellectual disabilities, including people with profound and multiple intellectual disabilities.

Rawles et al. (2025) retrospectively explored the experiences of people with milder intellectual disabilities about the COVID‐19 pandemic, as well as its long‐term impact. Participants with milder intellectual disabilities described how the pandemic disrupted their usual activities, which negatively impacted their mental wellbeing. Participants reported that families and third sector organisations helped to re‐establish access to social and leisure activities. The pandemic was particularly challenging for people living in supported living and residential environments, where social distancing and isolation led to feelings of frustration and where bereavements in the home led to feelings of grief, uncertainty and fear. Participants also reported being angry at the Government's handling of the pandemic. Some participants reported feeling optimistic about a future less impacted by public health restrictions. However, other participants expressed concerns about securing the right support and the cost of living.

Where research has focused on the experiences of the COVID‐19 pandemic for people with profound and multiple intellectual disabilities, it would appear that this group experienced more adverse outcomes (Flynn, Bailey, et al. 2021; Flynn, Hayden, et al. 2021a, 2021b; Hatton et al. 2024; Linden et al. 2022). Research has identified the challenges faced by families around balancing protecting the people they care for from infection with the impact of this on quality of life (Den Boer et al. 2023), the role of non‐governmental organisations in providing information and advice (Linden et al. 2022) and the impact of having health and social care services reduced or removed (Den Boer et al. 2023; Flynn, Bailey, et al. 2021; Flynn, Hayden, et al. 2021a, 2021b; Linden et al. 2022), such as increased isolation, deteriorating physical and mental health, increased behavioural signs of distress, and reduced life skills (Flynn, Bailey, et al. 2021; Flynn, Hayden, et al. 2021a, 2021b). Research findings have shown that towards the end of the pandemic, people with profound and multiple intellectual disabilities had poorer health and were less likely to be accessing health services than other people with intellectual disabilities (Hatton et al. 2024). When asked what would have made life better for people with profound and multiple intellectual disabilities during the pandemic family carers or paid support workers identified opportunities for social contact and activities, improved access to health and social care services, and consistent responsive staff (Bradshaw et al. 2024). Research findings have revealed some positive effects of the pandemic for this group, with family carers or paid support workers reporting that around half of the people with profound and multiple intellectual disabilities they cared for during the COVID‐19 pandemic found digital participation a new positive experience, with the potential to be developed further for some when restrictions eased (Caton et al. 2023).

There is scarce knowledge about the long‐term impacts of COVID‐19 for people with profound and multiple intellectual disabilities. The health and social inequalities experienced by people with profound and multiple intellectual disabilities are not new. However, it is likely that after years of austerity‐imposed erosion of health and social care services, a pandemic, and a cost‐of‐living crisis, inequalities have become more entrenched. New evidence may help us to build a richer picture of the pandemic's deeper impacts on this group and how we might best respond to these.

As part of the wider Coronavirus and People with Learning Disabilities Study (Flynn, Bailey, et al. 2021), we focused on the stories of people with profound and multiple intellectual disabilities as told through interviews carried out with family members. Three years after the pandemic started, and around 18 months on from pandemic‐related restrictions having been lifted, family members reflected back on what the pandemic had meant to their relative with a profound and multiple intellectual disability. Family members also reflected on what impact COVID‐19 was having on the present lives of their relatives, as well as their plans and hopes for the future. People with profound and multiple intellectual disabilities are a group who are often excluded from research (Boxall and Ralph 2011). Understanding what the pandemic meant and continues to mean to this group can highlight the current needs of people with profound and multiple intellectual disabilities, helping us to respond to future crises in ways that do not exacerbate existing inequalities for this group.

The research questions were as follows:

- After 3 years of the COVID‐19 pandemic, what has living through the COVID‐19 pandemic meant for people with profound and multiple intellectual disabilities and what does it continue to mean, as told through a family carer lens?
- What are the post‐pandemic plans and hopes for the future for people with profound and multiple intellectual disabilities, as told through a family carer lens?

## 2 Method

### 2.1 Research Approach

This was a qualitative research study using a narrative 2‐stage interview process.

### 2.2 Participants and Sampling

Purposive sampling was used to identify and invite 10 family members who took part in the Coronavirus and People with Learning Disabilities Study (Flynn, Bailey, et al. 2021) study. All of the family members who offered to take part were relatives of people with profound and multiple intellectual disabilities, which suggested that they felt more strongly about having a story to tell.

Family members of four men and three women with profound and multiple intellectual disability aged between 23 and 45 years (average age 39 years) participated in the study. Demographic information is summarised in Table 1.

**TABLE 1:** Demographic information of participants and relatives with profound and multiple intellectual disability.

Participant (Family member)	Sex of person with profound multiple intellectual disability	Age of Person with profound multiple intellectual disability	Living situation of person with profound multiple intellectual disability	Ethnicity of person with profound and multiple intellectual disability
1. Mother ‘Joy’	Female	45 years	Lived at home	White, British
2. Mother ‘Louise’	Male	42 years	Lived at home	Black, British
3. Sibling ‘Caitlin’	Male	40 years	Lived at home	White, British
4. Father ‘Thomas’	Male	40 years	Supported living	White, British
5. Mother ‘Jean’	Female daughter Female step‐daughter	41 years 41 years	Residential care Supported living	White, British White, British
6. Father ‘Ted’	Male	23 years	Supported living	White British

To protect their identity, participants are identified using pseudonyms.

### 2.3 Ethical Procedure

Research ethics approval was sought and obtained from Manchester Metropolitan University Ethics Committee.

Of those family members who agreed to take part, fully informed written consent was sought. Consent forms were emailed to family members who returned the forms before any interviews took place.

### 2.4 Interview Procedure

Ten potential participants were approached via email with details about the study. If they expressed an interest in taking part, they were sent information sheets, which included an offer of a voucher to thank participants for their time. Participants were also sent information about the research questions and a loose narrative interview schedule.

As taking part in the study entailed sharing in depth personal information about a potentially difficult period, an initial informal meeting was arranged with participants to talk over any questions they might have about their involvement and to establish rapport.

Participants took part in two interviews which took place between November 2022 and March 2023. Interview one was a narrative interview, whereupon family members were invited to tell their story. Each participant was asked questions on the same broad areas: what the life of the person with profound and multiple intellectual disability was like at the time of the first lockdown, what their life was like now and their hopes for the future. Participants were afforded as much freedom as possible in shaping and sharing their narrative. However, prompts were used if necessary, such as ‘Please tell me about X's week?’ or ‘Has anything changed for X?’

After the first interview, audio recordings were reviewed, and notes were made based on familiarisation drawings regarding any chronological gaps in the narrative or uncertainties that would benefit from further exploration in the second interview. These notes were used to produce a guide for the second interview, which took place one to 2 weeks after the first.

The aim of the second interview was to explore topics that arose in the first interview to deepen an understanding of people's experiences. A semi‐structured interview approach was used as it is a qualitative research method that allows researchers to ask participants pre‐determined open‐ended questions and probing follow‐up questions.

Participants were offered the option of taking part in the interviews in person. However, all participants opted to take part in online video interviews either on Zoom or MS Teams (depending on the preference of the participant). Interviews typically took around 90 min to complete.

Participants were interviewed by DC and JR on each occasion, both skilled interviewers with experience of carrying out qualitative interviews with people with intellectual disabilities and their families. At the end of the second interview, there was a debriefing conversation regarding the next steps of the study and to signpost to support services if necessary.

### 2.5 Data Analysis

This research was carried out using Polkinghorne's (1995) ‘analysis of narratives approach,’ where the data consist of narrative stories, but the data is analysed thematically. This was so that during the initial interview the interviewee had the freedom to tell their story in a way that was meaningful to them. According to Becker (1997) people construct stories to make sense of, and return order to, the disruptions they experience in life. By thematically analysing the content of these stories, we aimed to understand the ways in which people made sense of the pandemic and their present lives.

Interviews were audio recorded and transcribed by a professional transcription service. Braun and Clarke's (2021) thematic analysis approach were used to locate common themes among the narrative stories. First, familiarisation of each interview dataset was achieved through listening to interview audio recordings, reading and rereading interview transcripts, and producing familiarisation doodles, which were helpful for capturing ideas about potential patterns of meaning and any questions the researcher had. This meant being able to enter the coding phase of the transcription data with an engaged, critically questioning mindset.

The transcription data across all participants were then coded, starting with the data for interview one and then for interview two. Initially, themes were developed at an individual level: codes relating to each participant for transcripts one and two were reviewed and organised into themes, which captured the person's narrative, before moving on to the next case. The themes were then reviewed across all participants and developed to reflect the sample as a whole. The final themes were reviewed by the research team who agreed they accurately represented the stories told by participants in the interviews.

### 2.6 Quality Control Checks

Having colleagues carry out an independent audit of the analysis helped confirm the credibility of the analysis.

## 3 Results

The following section will present the themes and sub‐themes. These are outlined in Table 2.

**TABLE 2:** Main themes and sub‐themes.

Main theme	Sub‐theme
Theme 1. Deepening exclusion and inequality	*1:1 A history of exclusion and inequality*
	*1:2 ‘You're on your own’—Abandoned in pandemic response*
	*1:3 Lost services and support*
Theme 2. *‘*I still haven't recovered’—Exhausted by the pandemic.	—
Theme 3: A traumatic time—Disrupted routines, activities, and relationships	*3:1 Trauma vulnerability*
	*3:2 Disrupted routines and activities*
	*3:3 Relationships*
Theme 4: ‘The future is a scary place.’	—

### 3.1 Narrative Themes

#### 3.1.1 Theme 1: Deepening Exclusion and Inequality

A sense of deepening exclusion and inequality pervaded all the narrative accounts. Families who participated in the study reported that the pandemic, and the pandemic response, exacerbated pre‐existing health and social inequalities experienced by people with profound and multiple intellectual disabilities and that these led to new uncertainties post‐pandemic, especially around services and support.

##### 3.1.1.1 1:1 A History of Exclusion and Inequality

Families remarked on the ‘invisibility’ of people with profound and multiple intellectual disabilities, which can lead to them becoming overlooked when big policy decisions are made:

They're a very overlooked group. And it's because there's so few of them, they're still a minority within a minority. (Joy)

I think as a community, people with learning disabilities, but in particular people with high level support needs, with profound multiple disabilities, are very much invisible in society anyway. (Caitlin)

Families reported that before the pandemic they were struggling to obtain much‐needed services for their loved ones with profound and multiple intellectual disabilities after a decade of austerity and associated reductions in public funding for services:

All her stuff had stopped pretty much before COVID… She had her yoga, she had her music therapy… And there was Saturday Stories, and there was something else… They ran out of funding… And then COVID hit. (Joy)

##### 3.1.1.2 1:2 ‘You're on Your Own’—Abandoned in Pandemic Response

Families with relatives living at home remarked on how many of the services and supports they relied upon, such as respite support, were removed during the pandemic, leaving them having to care on their own and feeling abandoned:

We were not thought about at any point. The whole pandemic was much more stressful because it didn't feel like there was anyone remembering that we existed. (Caitlin)

The care was left to me. (Jean)

Families felt strongly that people with profound and multiple intellectual disabilities should have been protected and prioritised during the pandemic by the UK Government because of their higher risk of serious illness from COVID‐19. However, families reported that their loved ones were often overlooked or considered as an afterthought in pandemic response policy and planning when it came to issues such as personal protective equipment (PPE), vaccinations and shielding. This contributed to a sense of having to ‘fight the system’ due to concerns that they were being forgotten. For example, a key feature of the UK Government's guidance during the pandemic concerned shielding, giving advice to people identified as clinically extremely vulnerable from COVID‐19 to stay at home and to self‐isolate, with priority access to certain services. Families with loved ones living at home reported how their loved ones were not initially prioritised for shielding and how they had to battle for this to be changed:

He [The GP] just rang me up and he said, ‘X is not vulnerable, is she?’ And I said, ‘Well, yeah, of course she is, what do you mean?’ (Joy)

Shielding… we really had to fight for that to be recognised. (Caitlin)

Families described being unsure what to do about protecting their relative because of a lack of information from the UK Government and local authority. One family member reported the difficulties she encountered with trying to source personal protective equipment:

At the early stages I was ringing up saying, ‘What do I have to do about PPE? Where do I get PPE? How do I do it?’ Nobody could tell me; nobody had any idea… It was a bit like on the free market trying to find out myself. I did in the end, I sourced it. (Joy)

All family members remarked on the deepening discrimination experienced by people with profound and multiple intellectual disabilities during the pandemic. Of real concern to families was that people with profound and multiple intellectual disabilities would be excluded from lifesaving treatment if they were to fall seriously ill with COVID‐19. One family member described how angry she felt when a Do Not Attempt Cardiopulmonary Resuscitation Order (DNACPR) (NHS England 2021) was placed on her son's records without her knowledge when he was taken into hospital. She relayed how her son was suspected as having COVID‐19, but was later found to have a chest infection:

I was just angry and was able to challenge and say, ‘Who made the decision, on what basis?’… I said, ‘He's basically a healthy young man with some conditions, but he's not dying’… I knew it was a chest infection. They said it was COVID… I never thought that somebody would put a ‘Do Not Resuscitate’ without a discussion with me… If it had been my other son, I don't think we would have had that discussion. I thought it was dishonest and it was wrong on every level. (Louise)

Families continued to feel forgotten about in the pandemic response as restrictions were lifted. Families remarked that catching COVID‐19 could still be dangerous for their loved ones, even after being vaccinated, and that the UK Government failed to consider the liberties of people with learning disabilities in ending COVID‐19 restrictions. Families described having to rely on the personal choices of others to keep them safe, which left them feeling as if they were taking a huge risk every time they left the house:

What those people [The Government] don't realise is that those measures that were put in place were the things that made us start to think, maybe we can go to that farm shop, or maybe we can maybe go for lunch in that pub… Because we know that people are going to be wearing a mask… or we know that people are expected to stand two meters away. When they took all of those safeguards away, it actually sent us further back… and so we became more closed away. (Caitlin)

At the time of the second interview, a few families reported that their loved ones with profound and multiple intellectual disabilities had caught COVID‐19, but this had done nothing to alleviate their fears about what might happen should they catch it again. Families described how their loved ones faced a heightened risk due to physical health needs that are complex and that require high levels of support from others. Health issues such as having a compromised breathing system and/or dysphagia meant that their relative was more vulnerable to the virus and families were unsure how their relative might react to a different strain. Furthermore, having a profound and multiple intellectual disability also meant that their loved one found it difficult to respond to treatment, such as wearing an oxygen mask.

And so we just had to be grateful that that strain, or whatever, how it affected [him] that time didn't affect his breathing because that was the major thing we were concerned about. Because he has had hospital admissions for breathing difficulties, because he's not able to follow direction to cough it up or clear his throat or take a deep breath in, or able to tolerate having an oxygen mask on or anything like that. That was our biggest fear really. I am not less scared that COVID could potentially be fatal to [him] because [he] has had COVID before, the second time they've had COVID or however many times, it's not always milder and it can be different… (Caitlin)

…

Yeah, I am [worried] because… she can't cough… because what worries me, she's had it, she had COVID… hearing her all night… seeing the way she reacted to it… (Joy)

The need to protect their loved ones from the virus meant that these families were continuing to take measures to protect themselves, with one family wearing masks.

##### 3.1.1.3 1:3 Lost Services and Support

Participants reported that the pandemic saw the removal and scaling back of vital services including annual health checks, speech and language therapy, dentistry, physiotherapy, occupational therapy, day activity centres, respite care, hydrotherapy, and music therapy. Eighteen months on from the lifting of protections, families said that they were continuing to experience reduced or limited access to many services:

She used to have hydrotherapy on a Monday, but I don't think that's restarted. (Jean speaking about her daughter)

…

We haven't got physios, we can't recruit them. (Louise)

…

She's been on a waiting list for physio, OT, speech and language for over two years now. (Joy)

Some family members reported that this was having a negative impact on the health of the person with a profound and multiple intellectual disability. One family member reported that her daughter was non‐verbal, meaning that she could not communicate health symptoms easily. It had been 3 years since her daughter had an annual health check and she had concerns about her daughter putting on weight. Without a proactive approach to health to ensure effective ways of recognising and managing health conditions, families reported feeling that their relative's health needs were not being met, which placed additional care demands on them. Furthermore, there were concerns about how serious the implications for a person's health could be if health needs were not addressed, with the potential for deterioration and complications. For example, for one family member, a lack of access to physiotherapy for her son was viewed as leading to frailty, loss of function, loss of independence and a failure to recover after surgery:

I think it's affected him health‐wise. I think he's frailer, he can't do the things that he used to be able to do, like sit on the edge of the bed or sit up on his own. There's no reason why he can't get back to that, but he's going to need a lot of support and input and resources for that to happen. And considering that it's now nearly four months since he's had the operation and no physio whatsoever… they should be coming every week and making him sit on the edge of his bed, and things like that. (Louise)

Family members reported that the pandemic had also had an impact on the recruitment and retention of personal assistants. This left family members feeling like they were on their own and having to start all over again with training staff:

I had a good team of people that I'd employed just before lockdown… now I'm in a constant thing of trying to recruit… and it takes a long time to get to know someone like [my daughter] (Joy)

Families receiving individual budgets remarked that the amount they currently received was insufficient to meet the costs of recruiting new personal assistants, but that they dared not ask for more in case what they already had was taken away:

We are going to have to ask for more money for X's package because we can't recruit these new people and sustainably still pay. But everyone is so terrified of having money taken off them. (Caitlin)

Family members who had loved ones with profound and multiple intellectual disability living in residential or supported living arrangements described how the pandemic had made it more difficult to recruit and retain good quality staff, which was impacting on their loved one's quality of life. For example, one father reported that when the ‘better staff disappeared’ his son had a less active and enjoyable life because the people who used to actively engage with him were gone:

As COVID swept in some of the better staff disappeared… all the staff that tried to do things with him are gone. (Thomas)

#### 3.1.2 Theme 2: *‘*I Still Haven't Recovered’—Exhausted by the Pandemic

It was clear from all narrative accounts that the lives of family members and their loved ones with profound and multiple intellectual disability were inextricably interlinked. Therefore, while the focus of this research was on the lives of people with profound and multiple intellectual disabilities, the negative impact that the pandemic had on family member wellbeing was a clear theme across the interview data.

Feeling forgotten about in the pandemic response, and the closure and reduction in services and support, led to high levels of stress for family members, particularly those with loved ones with profound and multiple intellectual disabilities living at home:

I spoke to my doctor and said, ‘I can't do this anymore. I have no reserves left, none whatsoever.’ (Louise)

…

I stood at the bottom of the garden and cried in complete panic… I just looked ahead to the rest of the day and thought, I just cannot make it… I'm so exhausted, I feel so ill. (Caitlin)

Some people with profound and multiple intellectual disabilities lived in supported living or residential care. Family members who were separated from their loved ones due to visiting restrictions described feeling cut off from their loved ones, which meant that they were unable to check on their welfare and provide comfort during a distressing time. Families used to regularly visiting their children missed spending time with them. Families described the emotional pain of having been separated from their loved ones and described feeling traumatised by this:

But we didn't see [him] face‐to‐face for, probably, a couple of months. It's making me emotional now. The whole family has been traumatised… the effect on me has been absolutely traumatic. (Ted)

Some family members expressed concern for the long‐term impact of the pandemic on their physical and mental health:

I still think I haven't recovered from that physically. (Joy)

…

You know, I'm a 74‐year‐old geezer… that's one of the reasons why I found having [my son] at home at New Year more challenging than ever… I was exhausted by the COVID. (Thomas)

#### 3.1.3 Theme 3: A Traumatic Time—Disrupted Routines, Activities, and Relationships

Families reported that their loved ones with profound and multiple intellectual disabilities had experienced psychological trauma before the pandemic, rendering them more vulnerable to its negative effects. Families described how activities and routines, and relationships that provided structure and security, were severely disrupted. Throughout all stories, families perceived the daily lives of people with profound and multiple intellectual disabilities to continue to be impacted by the pandemic.

##### 3.1.3.1 3:1 Trauma Vulnerability

All families reported how loved ones with profound and multiple intellectual disabilities had a history of previous traumatic life experiences, including multiple placements, sudden changes to their living arrangements, experiences of abuse, parental separation, frequent and/or prolonged hospitalisations and bereavement.

A few people with profound and multiple intellectual disability went into the pandemic with reported ongoing psychological trauma from previous traumatic life events. For example, one family member described how the pandemic coincided with multiple losses for the person with profound and multiple intellectual disability:

So, she lost her mother, her home, her relationships, her ability to walk, her ability to eat, all within the space of two years, which is enormous for anybody, let alone for somebody who can't really understand why it's happening and can't articulate how they feel about it. And then COVID. (Jean, speaking about her stepdaughter)

For another person with profound and multiple intellectual disability, the start of the pandemic came at a time when there were significant problems with their health:

Right at the beginning of the lockdown, he was diagnosed as having a dislocated hip… Alongside that he went through a change in epilepsy medicine that sent him a bit odd. He got kidney stones. Then he had an operation on top of that. In between that, he got sepsis…. (Louise)

This left people with profound and multiple intellectual disabilities more vulnerable to the adverse effects of the pandemic on daily life:

Where he was at the time was so awful—and I mean it, seriously awful—that he also seemed to get quite depressed and resigned to the fact that that was what his life was going to be. (Ted)

Family members viewed the pandemic as an additional traumatic event in the lives of the person with profound and multiple intellectual disabilities:

So, the loss of family contact… it [the pandemic] was traumatic. (Ted)

Family members saw the pandemic as continuing to have a negative psychological impact on the mental wellbeing of the person with a profound and multiple intellectual disability. Family members describing ongoing trauma as manifesting through increased behaviours that challenge and mental health issues:

And we're getting very distressed behaviour at the moment because she's terrified of it [the pandemic] happening again. (Jean, speaking about her daughter)

##### 3.1.3.2 3:2 Disrupted Routines and Activities

Disruption to daily routines and activities during the pandemic featured throughout all the narrative accounts. Many of these activities had not resumed, or were operating at a reduced capacity at the time of the second interview, and were described as having physical and psychological consequences for the person with profound and multiple intellectual disability:

And, at the moment, there is no‐one to take him walking… And it's such a pity and certainly it will affect his fitness. I'm terrified he's going to get something like diabetes… (Thomas)

…

If you think 30 individuals basically would go and eat their lunch together. It was a fantastic little thing that went on once a week. It was [his]highlight of [the] week…. We'd have [cycling] races around the sports hall…. We know that all the people that used to go… have nowhere… for that activity now. (Ted)

Families with loved ones living in supported living or residential care reported that the quality of these settings appeared to be an important factor in whether people with profound and multiple intellectual disabilities had meaningful activity in their present lives:

He was so disappointed that year because there was nothing going on. There was no activity…. Yeah, so he does [have activities] now, but because he has moved, if he was still living in the same house, it would still be pretty nightmarish, I suspect, and very, very restricted in activities. (Ted)

…

Most of her time [my stepdaughter's] just in her home, with not a lot to do… Whereas [my daughter] has something on every day… The thing for me that has been highlighted, not just by the COVID, but in general, is the different ethos between the two different care settings. And I just think if one of them can be so brilliant, why can't that be rolled out to more places? (Jean)

Despite disrupted routines generally having a negative effect for people with profound and multiple intellectual disabilities, participants reported that there were some positive outcomes associated with the disruption. One family carer described how she and her brother enjoyed working on themed creative projects together:

So instead we had a theme for each day and some days took a lot of prep… I think day one was pond life and [my brother] and I made a complete edible pond. We had scrunched up gluten‐free biscuits for gravel and chocolate pudding for mud and jelly and I used little mandarin oranges for goldfish in the pond. (Caitlin)

Another family member reported how her daughter, who lived in a residential home, learned how to use Zoom during the pandemic and was continuing to use it to communicate with family members:

She did do really well mastering Zoom. I was very, very impressed how well she did with it. Her younger sister is on Zoom with her once a week on a Wednesday now. (Jean, speaking about her daughter)

##### 3.1.3.3 3:3 Relationships

Family members described how relationships were disrupted for people with profound and multiple intellectual disabilities because of the pandemic and that this negatively impacted their psychological well‐being.

For one family member who lived with her daughter in a flat, their relationship was put under intense strain during the pandemic, particularly during the first lockdown:

She's always had outbursts… but I've never seen this… it was like she was really angry… it was horrible, [she was] punching herself in the face so hard. (Joy)

Family members with relatives living in supported living or residential homes reported that their loved ones found the visitor restrictions particularly distressing. This was exacerbated in cases in which an individual was not able to, or was not supported, to have regular contact via internet access or phone calls Family members became emotional as they described how their loved ones struggled to understand what was happening, missed their families and the physical contact that they provided:

Not having physical contact, absolutely no hugs, and just ordinary… he's a six‐foot lad who regularly needs just a physical bear hug… He also needs physical compression across the shoulders. (Ted)

It was also reported that people with profound and multiple intellectual disabilities living in supported living or residential care were excluded from important family occasions, such as family weddings, Christmas celebrations and birthdays because they were not permitted to go home:

I think that if COVID… well, for a start they would have both been able to come to our wedding, which neither of them could. (Jean)

One family member remarked that they felt as if they were being deliberately prevented from seeing their loved one as restrictions lifted. This same family member reported that the visitor regulations increased his son's susceptibility to abuse by the member of staff who sought to isolate him:

This care worker had prevented, on multiple occasions, family contact… saying that ‘[My son] has said he's not in the right frame of mind.’ That happened countless times… We found out that when he was on his own with [him]… he stood over and berated him into his face, shouting, ‘You, effing retard.’… Over a period of time, the regulations of lockdown had enabled this care worker to ring‐fence [him] and prevent him having much contact with other people that would see that he was being mistreated. (Ted)

Some families reported that they felt that the pandemic and the steps taken to mitigate the disease, such as visitor restrictions, had adversely changed their loved ones:

And the other thing, he's a lot more assertive now than he was three years ago… and mostly he's assertive in things he doesn't want to do. (Thomas)

…

The pandemic was quite significant for him, so he has become much more anxious… it has never quite gone back. (Ted)

…

She had a very traumatic Christmas when she wasn't allowed to come home and that really distressed her. (Jean, speaking about her daughter)

A few family members reported that relationships had not recovered:

And then, when the pandemic came, I was only ever seen on the road… So, my relationship with [him] changed fundamentally, which was really sad for me and for [him]. And it's never recovered fully, even to this day. (Ted)

#### 3.1.4 Theme 4: ‘The Future Is a Scary Place’

Throughout all the stories, family members expressed much anxiety about the future of their loved ones with a profound and multiple intellectual disability:

The future is a scary place, constantly trying to work out levels of support. (Caitlin)

With the exception of one family member, all participants were nearing retirement or had already retired and feared what would happen to their loved one if they were unable to care for them or had passed away. Some families with loved ones living at home feared that their loved ones would be institutionalised:

I think I mentioned before how averse I am to residential stuff… I could never—I would never—have chosen it for [him]. (Louise)

Families with loved ones living in supported living and residential care feared that the standard of care would drop and/or that their loved one would be at risk of further institutionalisation if they were not there to fight for them:

The concern also we have is that the quality of care that's delivered to him, which is good at the moment… If that changed… that would be of huge concern… whether we're around or not. (Ted)

Although these anxieties had been in the background for some time, the COVID‐19 pandemic appeared to make families more aware of the need for thinking about the future and long‐term care planning:

So COVID was this very insular world… I can remember looking at us and thinking, he's in his early 40s and the people looking after him are these old ladies. I just thought, oh, this isn't right… but as it has evolved and because of COVID… I [had] imagined that he would be living here with us… but looking in the long‐term, there's no reason why [he] won't outlive us. (Louise)

## 4 Discussion

This study aimed to explore what living through the COVID‐19 pandemic meant for people with profound and multiple intellectual disabilities, what it continues to mean and hopes for the future as told through a family carer lens. The research findings show that the pandemic had a significant impact on the lives of people with profound and multiple intellectual disabilities. Participants reported that they felt as if their relatives with profound and multiple intellectual disabilities were not prioritised for shielding and vaccination, despite early evidence that they had a high risk and morbidity from the disease (Henderson et al. 2022). Furthermore, they felt that there was a lack of guidance from the UK Government about whether families of people with profound and multiple intellectual disabilities could continue to have support from personal assistants and how they could make their homes COVID‐19 secure. This meant that families stopped employing personal assistants, leaving them to fend for themselves. These findings are consistent with other research that shows that Government inaction and indifference exacerbated the vulnerability of people with intellectual disabilities during the pandemic, putting people's lives and wellbeing at risk (Scherer et al. 2023). Families reported feeling thoroughly exhausted as the burden of care fell primarily on them. This adds to the body of evidence demonstrating the negative impact of the pandemic on the mental wellbeing of family carers of people with profound and multiple intellectual disabilities (Flynn, Bailey, et al. 2021; Flynn, Hayden, et al. 2021a, 2021b; Linden et al. 2022).

Family members reported that many health appointments, therapies, as well as day care and respite services were cancelled, delayed or moved online during the pandemic, putting their relative's physical and mental wellbeing at risk. Many organised activities (e.g., hiking or swimming) that provided structure, routine, security, stimulation, and connections were also severely disrupted. This also lends support to evidence from previous studies that show that services were cut or scaled back (Flynn, Bailey, et al. 2021; Flynn, Hayden, et al. 2021a, 2021b; Rawles et al. 2025; Scherer et al. 2023).

The COVID‐19 pandemic was traumatic for many people, but particularly for people with intellectual disability, who are likely to experience more adverse life events and abuse than the general population (McNally et al. 2021). There is a gap in the research literature regarding trauma in the lives of people with profound and multiple intellectual disability, including literature exploring the experiences of this group during the COVID‐19 pandemic. A novel insight from this study is that family members perceived the pandemic to have been a traumatic event in the lives of their relative with a profound and multiple intellectual disability, with long‐term impacts on their mental health (e.g., increased anxiety). For people with profound and multiple intellectual disabilities, who can find it difficult to understand what is being communicated to them, visiting restrictions were particularly confusing and distressing. For a few families, this damaged relationships with their relatives that as yet showed no signs of repair. Participants also relayed that the pandemic was a traumatic experience for them. This might help explain why at interview participants were overly focused on past events, meaning that the data was less rich regarding what the lives of their relatives were like now.

While it was reported that people with profound and multiple intellectual disabilities mainly had negative experiences of the pandemic, one family member relayed that their daughter developed digital skills so as to communicate with their family during visiting restrictions. The addition of digital participation in their daughter's life was purported to be beneficial enough for them to be continuing to use online devices at the time of the second interview. In previous research by Caton et al. (2023) 91 family carers or paid support workers of adults with profound and intellectual disabilities completed an online survey about the experiences of the person they cared for during the COVID‐19 pandemic. This included questions on internet use and found that around half of people surveyed participated in digital activities. Caton et al. (2023) also found that there was a need to create opportunities for digital participation and for more support with technology use for this group, especially as new ways of communication and new experiences can take time for people with profound and multiple learning disabilities to get used to. It would be useful for future research to explore how people with profound and multiple intellectual disabilities currently engage with technology and whether this has changed since the pandemic.

The findings from this study have significance relevance for present times. With the arrival of vaccines and the end of restrictions it would be easy to assume that the impact of COVID‐19 has come to an end. However, it has been argued that ‘we are in a COVID decade’ and that the societal impacts of COVID‐19 will have profound effects upon the UK for many years to come (British Academy 2021). The pandemic has exposed and exacerbated pre‐existing inequalities and inequities which has meant that groups such as people with profound and multiple intellectual disabilities are more vulnerable than before (Scherer et al. 2023). For example, challenges accessing health care services continue, with health inequalities persisting (The Care Quality Commission 2024). This is exemplified by the research findings, which show that the pandemic continued to impact participants' daily lives. Some family members reported that around 18 months after the removal of COVID‐19 protections they were continuing to encounter problems with accessing these services and that this was having an ongoing negative impact on their relative's health. This finding is supported by data from the UK Coronavirus Study which shows that by wave 4 (autumn 2022, over a year after public health protections stopped) people with profound and multiple intellectual disabilities had poorer health, but were not accessing health services as much as other people with intellectual disabilities (Hatton et al. 2024).

A new insight from this study is that the ongoing impact of not being able to access services may have been greater for people with profound and multiple intellectual disabilities. Unlike people with milder intellectual disabilities, people with profound and multiple intellectual disabilities are at increased risk of experiencing multiple and complex health conditions, which require ongoing monitoring and management (van Timmeren et al. 2017). This means that when services essential for continued health and wellbeing are removed, the impact is likely to be more keenly felt. This was illustrated by the finding from this study that the removal of healthcare services such as physiotherapy had resulted in the development of further health complications and deterioration for a person with a profound and multiple intellectual disability, placing additional strain on the family member caring for them.

At the time of the second interview a few families reported to continue to fear their relatives catching COVID‐19 and were continuing to take measures to protect them from the virus. While COVID‐19 is no longer categorised as a Public Health Emergency of International Concern (PHEIC), it is still very much a global threat (Geddes 2023). The virus is here to stay, and it continues to mutate. On 1st October 2024, the total number of hospital beds in England occupied by confirmed COVID‐19 patients was 2069 (NHS England, n.d.). Although the monthly data that NHS England collects on the total number of beds occupied by confirmed COVID‐19 cases does not tell us whether this includes people with profound and multiple intellectual disabilities, we know that COVID‐19 poses a particular risk for this group.

Participants reported that for some people with profound and multiple intellectual disabilities many organised activities had not resumed or were operating at a reduced capacity. This was reported to have had an ongoing negative effect on both the physical and mental wellbeing of people with profound and multiple intellectual disabilities. Unlike people with milder intellectual disabilities, however, reduced access to activities was found to impact negatively on previously acquired skills. Assessment may help to identify the areas where there has been a regression and where targeted support is required.

Some family members reported that the pandemic had made them more aware that they were getting older, and that caring was becoming more difficult. This, in turn, strengthened fears about the future and the need for long‐term care planning. A Dutch study also found that awareness of long‐term care planning grew during the pandemic among family carers of people with intellectual disabilities (Boeije et al. 2023). However, while awareness moved some family members to action (Boeije et al. 2023), the family members who took part in this study reported that they did not know anyone they could trust to take over their care tasks. These feelings may have been exacerbated by the ongoing struggles they appeared to have with finding competent staff due to issues around staff retention and recruitment, and the crisis in social care. Future research could focus specifically on exploring the experiences of family carers of people with profound and multiple intellectual disabilities when thinking about and making arrangements for when they are no longer able or willing to provide care and support themselves.

It is important to note that people with profound and multiple intellectual disabilities are a much smaller group within the intellectual disability population and are rarely considered in policy that is aimed at people with intellectual disabilities at both a national and local level As Lyle (2019) states, ‘oftentimes policymakers and commissioners of services have little direct knowledge of these people. Or even if they do, will overlook them in order to promote the group characteristics, which better fit their policy (p. x)’ There is an urgent need going forward to consult with people with profound and multiple intellectual disabilities and their families so that services and supports impacted by the pandemic are not just reinstated, but also improved. It is especially important that people with profound and multiple intellectual disabilities be able to access good health services because of their extensive needs for healthcare and support (Van Timmeren et al. 2016). Consultation is also important to ensure that in the event of another pandemic people with profound and multiple disabilities are prioritised. Preventing traumatisation, re‐traumatisation and promoting healing will be key to helping people with profound and multiple intellectual disabilities repair from the traumatising effects of the pandemic and future research should focus on how this might be achieved. In recent years, there has been a drive within the UK for organisations providing supported living and residential services to become more trauma‐informed and trauma responsive (McNally 2022). The findings from this study reinforce the imperative to maintain and strengthen trauma‐informed policies and practices.

According to Nind (2008) ‘For some people whose learning difficulties are more profound, no amount of visual or other structure will make the interview method possible’ (p. 11). In such cases, it is usual to identify a significant other (proxy) who knows the person well and can ‘speak’ on their behalf. For the current study, significant others were family members such as parents or siblings, who had self‐defined as ‘knowing the person well’ as part of the wider study. Boxall and Ralph (2009) state that using proxies provides a useful means of getting close to the perspective and voices of people with profound and multiple intellectual disabilities. Family members as proxies are likely to have spent enough time with the person to be able to accurately describe their life experience‐their likes and dislikes, the level of support they require, and so forth.

All family members appeared to know the person with profound and multiple intellectual disability well. However, we can never assume that family members know everything about their relative, especially in cases where the person with profound and multiple intellectual disability lives in separate accommodation. Cluley (2016) also points out that because of the communication and cognitive difficulties inherent in people with profound and multiple intellectual disabilities, family members often have to make presumptions about the experiences of the people that they care for based on their relationship and knowledge of them. So, while the views of family members are important, they must also be interpreted with care because we cannot be certain that they are accurately representing the experiences of people with profound and multiple intellectual disabilities. As family members lives were invariably interlinked with their relative, research team discussions helped to determine what aspects of the family member experience might be useful to include in the analysis, whilst ensuring that the lives of people with profound and multiple intellectual disabilities remained the primary focus.

Finally, as people with profound and multiple intellectual disabilities are a minority group, which was reflected in the small number of family members who were available for recruitment from the wider study, there were some limitations to the extent to which a diverse sample could be attained. For example, all but one of the family members recruited to the study were of retirement age, with relatives with profound and multiple intellectual disabilities in their 40s. A strength of the study, however, is that it captures the different living circumstances of people with profound and multiple intellectual disabilities, which has implications for how the pandemic impacted both their past and present lives.

## Author Contributions

**Dawn E. Cavanagh:** wrote the paper, collected the data, performed the analysis, conceived and designed the analysis. **Sue Caton:** conceptualisation, funding acquisition, conceived and designed the analysis, wrote the paper. **Jodie Rawles:** collected the data, performed the analysis, conceived and designed the analysis, wrote the paper. **Chris Hatton:** conceptualisation, funding acquisition, conceived and designed the analysis, wrote the paper. **Richard P. Hastings:** conceptualisation, funding acquisition, wrote the paper.

## Conflicts of Interest

The authors declare no conflicts of interest.

## Data Availability

Research data are not shared.
